# The eTRANSAFE Project on Translational Safety Assessment through Integrative Knowledge Management: Achievements and Perspectives

**DOI:** 10.3390/ph14030237

**Published:** 2021-03-08

**Authors:** François Pognan, Thomas Steger-Hartmann, Carlos Díaz, Niklas Blomberg, Frank Bringezu, Katharine Briggs, Giulia Callegaro, Salvador Capella-Gutierrez, Emilio Centeno, Javier Corvi, Philip Drew, William C. Drewe, José M. Fernández, Laura I. Furlong, Emre Guney, Jan A. Kors, Miguel Angel Mayer, Manuel Pastor, Janet Piñero, Juan Manuel Ramírez-Anguita, Francesco Ronzano, Philip Rowell, Josep Saüch-Pitarch, Alfonso Valencia, Bob van de Water, Johan van der Lei, Erik van Mulligen, Ferran Sanz

**Affiliations:** 1 Preclinical Safety/Translational Medicine, Novartis, 4057 Basel, Switzerland; francois.pognan@novartis.com; 2 Pharmaceuticals, Investigational Toxicology, Bayer AG, 13342 Berlin, Germany; thomas.steger-hartmann@bayer.com; 3 Synapse Research Managers SL, 28006 Madrid, Spain; cdiaz@synapse-managers.com; 4 ELIXIR, Hinxton, Cambridgeshire CB10 1SD, UK; niklas.blomberg@elixir-europe.org; 5 Chemical & Preclinical Safety, Merck Healthcare KGaA, 64293 Darmstadt, Germany; frank.bringezu@merckgroup.com; 6 Lhasa Limited, Leeds LS11 5PS, UK; katharine.briggs@lhasalimited.org (K.B.); Will.Drewe@lhasalimited.org (W.C.D.); phil.rowell@lhasalimited.org (P.R.); 7 Leiden Academic Centre for Drug Research (LACDR), Leiden University, 2300 RA Leiden, The Netherlands; g.callegaro@lacdr.leidenuniv.nl (G.C.); b.water@lacdr.leidenuniv.nl (B.v.d.W.); 8 Barcelona Supercomputing Center (BSC), 08034 Barcelona, Spain; salvador.capella@bsc.es (S.C.-G.); javier.corvi@bsc.es (J.C.); jose.m.fernandez@bsc.es (J.M.F.); alfonso.valencia@bsc.es (A.V.); 9 GRIB, Hospital del Mar Institute of Medical Research (IMIM), DCEXS, Pompeu Fabra University (UPF), 08003 Barcelona, Spain; ecenteno@imim.es (E.C.); laura.furlong@upf.edu (L.I.F.); emre.guney@upf.edu (E.G.); miguelangel.mayer@upf.edu (M.A.M.); manuel.pastor@upf.edu (M.P.); janet.pinero@upf.edu (J.P.); palilson.jmr@gmail.com (J.M.R.-A.); francesco.ronzano@upf.edu (F.R.); jsauch@imim.es (J.S.-P.); 10 PDS Consultants, Leicester LE1 5XY, UK; philip.drew@pds-consultants.co.uk; 11 MedBioinformatics Solutions SL, 08018 Barcelona, Spain; 12 Department of Medical Informatics, Erasmus University Medical Center, 3015 GD Rotterdam, The Netherlands; j.kors@erasmusmc.nl (J.A.K.); j.vanderlei@erasmusmc.nl (J.v.d.L.); e.vanmulligen@erasmusmc.nl (E.v.M.); 13 Catalan Institution for Research and Advanced Studies (ICREA), 08010 Barcelona, Spain

**Keywords:** toxicology, drug safety, translational safety assessment, data sharing, integrative knowledge management, data mining, read across, predictive modelling

## Abstract

eTRANSAFE is a research project funded within the Innovative Medicines Initiative (IMI), which aims at developing integrated databases and computational tools (the eTRANSAFE ToxHub) that support the translational safety assessment of new drugs by using legacy data provided by the pharmaceutical companies that participate in the project. The project objectives include the development of databases containing preclinical and clinical data, computational systems for translational analysis including tools for data query, analysis and visualization, as well as computational models to explain and predict drug safety events.

## 1. Introduction

Drug discovery and development is a knowledge-intensive process that can benefit from integrative analysis of data of different types and sources. These integrative approaches can facilitate decision-making along the drug discovery and development process, improving lead optimization and enhancing drug safety. A prerequisite for this is the availability of relevant, high-quality datasets [1], such as the ones accumulated in the archives of the pharmaceutical companies. However, to maximally leverage these data, we face important challenges, such as encouraging information sharing among competing organizations, or promoting adequate data standardization, annotation and quality control [2]. The eTOX project on legacy data sharing for predictive toxicology constituted a cornerstone in the processes of data sharing and integrative analysis for improving drug toxicity prediction [3]. In addition to collecting and integrating an unprecedented amount of toxicological data from the pharma industry (nearly nine million preclinical data points corresponding to 8196 toxicity studies carried out on 1947 compounds), eTOX provided first-hand experience on the aforementioned challenges [3,4], as well as those related to the development of predictive models for in vivo toxicological outcomes [5].

**Figure 1:**
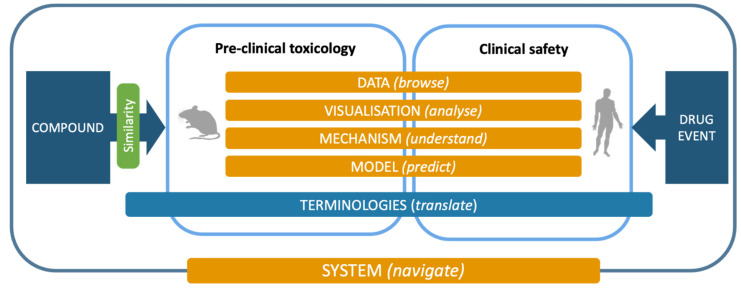
The eTRANSAFE concept, components and processes.

Following but extending the objectives of the eTOX project, the ongoing eTRANSAFE project is focused on the challenging field of translational safety evaluation and aims to provide in silico solutions for identifying when and how much the preclinical toxicological observations can be predictive of clinical adverse drug reactions. The eTRANSAFE project has an ambitious scope of objectives, which include the development of preclinical and clinical databases on the basis of legacy information, computational systems for translational analysis, including tools for data query, analysis and visualization, as well as in silico models to explain and predict drug safety events (see Table 1). The concept, components and tools of the project are depicted in Figure 1.

## 2. Translational Safety Assessment: Concept, Challenges and Semantic Tools

Regulatory preclinical studies are performed to enable safety margin calculations over expected human exposures and allow a new drug candidate to be first tested in healthy volunteers and thereafter in a number of patients for safety and efficacy assessments. This legally enforced methodology is commonly accepted as the least risky procedure to test a completely new drug modality in humans. Conceptually, this strategy is straightforward, with a number of accepted assumptions and empirical data permitting a relatively informed estimation of what untoward events might be expected in humans. However, there are several challenges and difficulties in the translation of animal data for the assessment of human safety, such as:

- Rats, rabbits, dogs and all other preclinical species show non-negligible differences in their biology among them and compared to humans (e.g., receptor affinities, enzymatic activities, role of hormones, growth chronology, food regimens). Hence, the action of a drug on the physiology of animals will not be a true indication of the possible effects in humans.
- For the same reasons, the processes of pharmacokinetics, metabolism and elimination of drugs will also differ between species.
- Beyond physiology, the morphology of the mammalian species also differs, with rats, for example, being devoid of gallbladder but having a forestomach and an extra periorbital organ, the harderian gland. Moreover, preclinical animals, with the exception of the mini pig, are furry and the translation to humans of preclinical skin toxicity assessment is known to be one of the poorest.
- Preclinical animals are also young, in controlled good health, thoroughly monitored and tested before the study starts, and they are mostly inbred, i.e., of high genetic homogeneity. These are striking differences with the diverse human population in need of drug treatments.
- The procedures for preclinical toxicity assessment widely differ from clinical practices. Animal studies are usually carried out at high doses, ending in full body necropsy with organ gross pathology, organ weight determination, thorough histopathological assessment with several types of staining, full haematology and comprehensive clinical chemistry, along with clinical observations, body weight, food intake, cardiovascular investigations and more specialized endpoints during the in-live phase of the studies. Non-invasive imaging technologies may also come to use. The organ and tissue histopathology assessment hereby represents the core of the toxicology study reports. On the human side, autopsies or biopsies to access tissues of interest are rarely performed, restricting the assessment in clinical trials to clinical observation and interpretation of symptoms. Additional imaging techniques may sometimes supplement this diagnostic approach, with blood analyses for haematology and clinical chemistry completing the clinical toolbox. Hence, clinical chemistry, cardiovascular parameters and haematology would be the most straightforward translational aspect between animals and humans; the human symptom descriptions would find their rough equivalents in animal clinical observations; while the almost exhaustive list of organs and tissues observed by histopathology in animals would find only a scarce set of selected human biopsy counterparts.
- Last and perhaps one of the most challenging aspects is the difference in vocabulary used on each side of the equation. This is coming from the aforementioned differences, but also because the preclinical and clinical worlds use their own technical and professional terminologies.

For the consideration of progressing one new compound into humans, all these difficulties are addressed by the expertise and practices of professionals on both the preclinical and clinical sides. However, if one wants to cope with the global paradigm and extract unearthed knowledge out of the wealth of accumulated data on both sides, and analyse the true predictive power of animal studies, a general correspondence, or translation, of these two universes has to be made. This is the core aim of the eTRANSAFE project. Previous attempts at assessing how animal data can be predictive of human safety have been published and set a good foundation [6]. Until recently [7], most of these analyses were limited to only a few preclinical studies, usually considering sensitive proprietary data only available to the authors. Here, using the previous eTOX preclinical database, and the eTRANSAFE preclinical and clinical databases, a systematic and thorough analysis can be made on a global scale.

**Figure 2:**
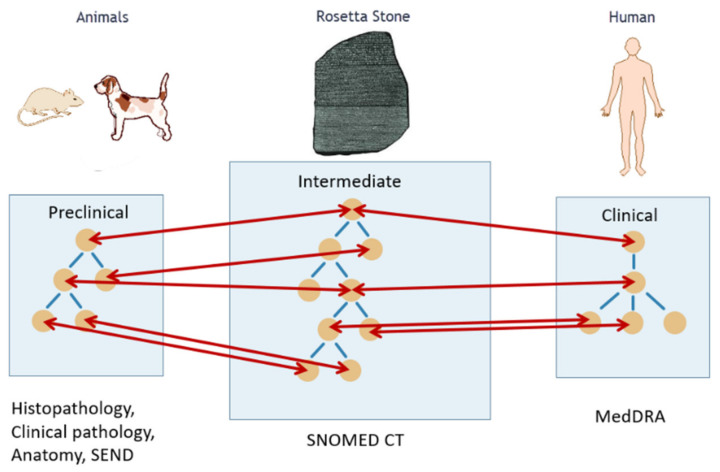
Translation between animal and human ontologies using a common intermediate, the Rosetta Stone, for safety assessment.

A key activity of the eTRANSAFE project in this respect has been the development of a mapping between preclinical (e.g., SEND) and clinical (e.g., MedDRA) ontologies using a common intermediate (SNOMED CT) by applying the Rosetta Stone strategy (Figure 2). This mapping has been implemented in a computational tool that provides semantic services to the eTRANSAFE system.

## 3. Preclinical Legacy Data Available in eTRANSAFE.

**Figure 3:**
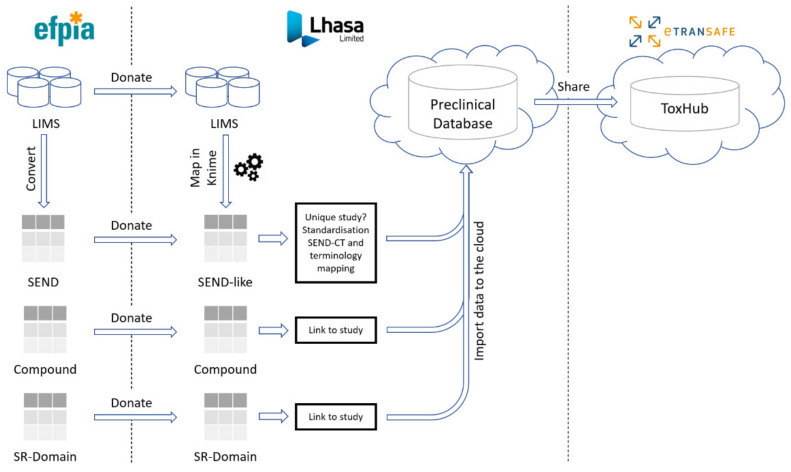
The preclinical data pipeline in eTRANSAFE.

Preclinical data are shared in eTRANSAFE through the cloud-hosted Preclinical Database Platform, designed and developed by Lhasa Limited, the preclinical data honest broker. These data comprise in vivo animal toxicology study data, compound information including off-target pharmacology data and the novel Study Report (SR) Domain data (Figure 3).

### 3.1. In Vivo Animal Toxicology Study Data 

The eTRANSAFE in vivo animal toxicology data sharing is centred on the CDISC Standard for Exchange of Non-Clinical Data (SEND) format, which was mandated by the FDA as the standard to be used for new drug submissions from December 2016. Where available, the pharmaceutical partners of eTRANSAFE donate these toxicological data in SEND format for importation into the Preclinical Database Platform. Where these data reside in Laboratory Information Management Systems (LIMS) in a non-SEND format, these data require their mapping to the standardized SEND-like schema of the Preclinical Database Platform using mapping rules developed using KNIME [8].

Further in vivo animal toxicology study data have been made available through the integration of 6500 preclinical studies from the eTOX project [3] into the eTRANSAFE ToxHub System. This enables eTRANSAFE to capitalize on and sustain the data sharing efforts resulting from the eTOX project.

### 3.2. Compound Information

Compound information comprises compound specific data, such as the chemical structure (which is not part of SEND), chemical name(s) and CAS registry number. In addition, data originating from in vitro off-target screening assays are considered within this data category. To include these data within the Preclinical Database Platform, a database table was designed based on representative EFPIA off-target screening assay datasets. To date, off-target screening assay data for 539 compounds have been donated by eTRANSAFE pharmaceutical partners. Recently, EMBL-EBI has investigated the potential to apply the standardization methodologies applied to the ChEMBL database [9] to the shared EFPIA off-target data to better facilitate model development. These standardized off-target screening assay data will be integrated into the ToxHub platform.

### 3.3. Study Report (SR) Domain 

To enhance the usefulness of the EFPIA preclinical data shared in eTRANSAFE, it is essential to capture the expert conclusions of animal toxicology studies in a consistent, structured, machine-readable format. Such expert conclusions contain observations for risk, severity and statistical significance qualified by the group, sex, day, specimen and test to which they relate. These expert conclusions are not captured in SEND. To achieve this goal, PDS Consultants have developed the concept of the Study Report (SR) Domain as a proposed additional domain for SEND. The proposed SR Domain has been integrated into the eTRANSAFE preclinical database schema to enable such data to be captured and utilized. Currently, the eTRANSAFE consortium is investigating methods to populate the SR Domain. These methods include the manual extraction of study report data using a computer interface specifically developed to facilitate this task, in addition to text mining expert findings from study reports (see the corresponding section of this article). The SR Domain application may also enable Study Directors to record significant study findings directly within an SR Domain upon the conclusion of a study.

### 3.4. The eTRANSAFE Preclinical Database Platform

The eTRANSAFE Preclinical Database Platform has been developed by Lhasa Limited as a cloud-hosted platform designed to securely store and share EFPIA SEND, mapped LIMS (non-SEND), compound and SR Domain preclinical data (Figure 3). The schema of this database is centred on the standard SEND domain tables, with additional tables for the SR Domain and compound data. Data are stored in the original (raw) form and are also standardized to the SEND controlled terminology (SEND-CT). Due to the limited scope of the SEND-CT, standardization of terms using other ontologies, such as the eTOX ontology [10], is envisaged. The imported, standardised data are made available to the eTRANSAFE ToxHub platform via a primitive adaptor component which has been designed to provide data from several database resources in a uniform format for querying.

### 3.5. Preclinical Data Sharing Statistics

**Figure 4:**
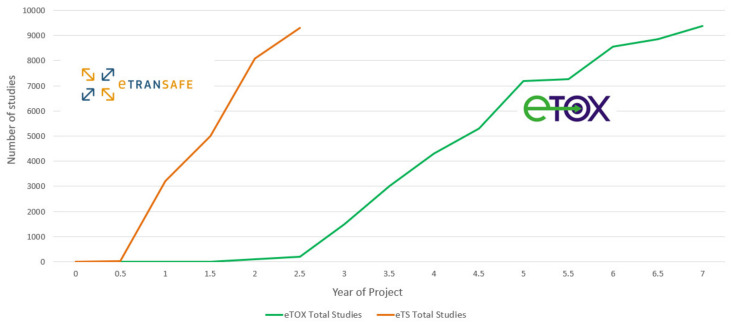
The number of study donations made to the eTOX and eTRANSAFE projects over time.

A summary of the preclinical data donated by eTRANSAFE EFPIA at the mid-point of the eTRANSAFE project is shown in Figure 4. This illustrates that relative to the data donated in the eTOX project, the pace of data donation to this point in eTRANSAFE is significantly accelerated. However, there remain challenges for pharma companies when sharing preclinical data in projects such as eTRANSAFE, the most significant of which relates to overcoming the legal barriers for fully sharing these data with the other project partners. Of the data donated to this point, 91% are classified as data which cannot be shared within the project consortium. To overcome this barrier, the partners of the eTRANSAFE project are exploring opportunities to enable “partial data sharing” of preclinical study data which would otherwise not be sharable. Such “partially sharable data” categorization enables the sharing of all data contained within an in vivo toxicological study but excludes the sharing of compound information such as the chemical structure, internal code, name or reference, the pharmacological target, the indication(s), data from the off-target in vitro assay screening panel and the company name or identifier. It is anticipated that by enabling “partial data sharing” in eTRANSAFE, the legal barriers for data sharing are reduced, enabling an increased volume of preclinical data to be made available for contributing to the translational objectives of eTRANSAFE.

The value of the study data for which the compound information is not shared is four-fold: first, the control group data from such studies can be used for calculating reference or control value ranges for various parameters, which can also be used for constructing virtual control groups (see the specific section in this article). Second, if, during a query for specific toxicological findings data, they are found in a partially shared study, there remains always the possibility to contact the study provider, enquiring as to whether additional information can be shared on a 1:1 confidential basis. This might be of particular interest if the query is related to rare findings. Third, meta-analyses on the occurrence of findings and their respective species sensitivities do not require chemical structures. Fourth, the partially shared data should gradually move to fully shared data as their sensitivity for the data owner diminishes with time. In this regard, we believe that it is better to already bank the non-shareable data and with time simply modify their status, rather than acquiring them only after status change.

## 4. Clinical Data

The clinical data in eTRANSAFE comprise data from publicly available resources and from eTRANSAFE EFPIA partners.

### 4.1. Clinical Data Sharing Statistics

For data in the public domain, adverse drug reactions were collected from three different types of resources: clinical trial registries, spontaneous reporting systems and the scientific literature.

For clinical trials, a broad range of clinical trial data registries exist. In eTRANSAFE, data were gathered from ClinicalTrials.gov, one of the largest and oldest registries containing information about publicly and privately funded clinical studies conducted around the world. The data from ClinicalTrials.gov were downloaded and post-processed to make the data fit for inclusion in the ToxHub database. For this postprocessing, the Sherlock system was used [11]. Sherlock converts the XML files that can be downloaded from ClinicalTrials.gov into a relational database and normalizes adverse reactions and drugs to concepts from the Medical Dictionary for Regulatory Activities (MedDRA) and the RxNorm terminology, respectively.

Spontaneous reporting systems are a cornerstone for drug safety surveillance during the post-marketing phase of drugs. In eTRANSAFE, spontaneous reports were taken from the publicly available Federal Drug Administration (FDA) Adverse Event Reporting System (FAERS). The data from FAERS need standardization before they can be used in the Toxhub system. For this, we used the AEOLUS system [12]. AEOLUS removes duplicate case records, standardizes drug names and outcomes to RxNorm and MedDRA concepts and computes summary statistics about drug–outcome relationships, such as the proportional reporting ratio.

The published biomedical literature also contains a host of information about adverse drug reactions. Several researchers have proposed methods to mine MEDLINE, the largest biomedical literature repository, for adverse events using the indexing of MEDLINE publications with Medical Subject Headings (MeSH). In eTRANSAFE, we use the method proposed by Winnenburg et al. [13], which was shown to yield a significant improvement over a baseline approach [14]. We use the Unified Medical Language System (UMLS) to map the MeSH terms to MedDRA concepts before inclusion in the eTRANSAFE data repository.

### 4.2. The Periodic Safety Update Reports Database

The objective of the Periodic Safety Update Reports (PSURs) generated by the pharmaceutical companies is to provide an extensive and critical analysis of the risk–benefit balance of medicinal products (MP) for the regulatory authorities. The PSUR constitutes a tool for the post-authorization evaluation at different moments in the lifecycle of an MP and provides summarized quantitative information on the adverse health events observed during clinical trials and pharmacovigilance. In order to exploit the drug safety information included in the PSURs, we have created a database based on the PSURs provided by the eTRANSAFE pharma partners. The eTRANSAFE PSUR database consists of three sets of tables. The first one includes the description of the MP, the period of validity of the report, the type of data stored and the pharmaceutical company. In the second set, data on the number of times that specific adverse effects have been observed after using a MP are stored. The third set of tables contains the MedDRA vocabulary for the proper definition of the adverse effects observed. To facilitate the access to this database and the search of information, a user-friendly web-based graphical interface has been developed, providing diverse capabilities for exploring, filtering and ranking the information. The interface also includes a functionality for downloading the information resulting from the queries. In the framework of the integration of the PSUR database into the eTRANSAFE ToxHub, additional functionalities will be incorporated, such as more sophisticated querying capabilities and the connection to the other databases.

## 5. Text Mining for Key Data Gathering

Over the last few decades, the pharmaceutical industry has generated a vast corpus of knowledge on the safety and efficacy of investigational drugs based on preclinical model systems. Despite the potential value of this knowledge to support decision-making in the drug development process, the fact that most of these data are only available as unstructured textual documents with variable degrees of digitization has hampered their systematic access, use and exploitation. This limitation can be avoided, or at least mitigated, by relying on text mining techniques for automatically extracting relevant, structured data from textual documents of interest, thus supporting drug research and development activities.

Toxicology reports describing the results of preclinical toxicology studies carried out by pharmaceutical companies have been identified as a valuable source of information on safety findings for investigational drugs in the context of the eTRANSAFE project. However, the exploitation of the preclinical knowledge contained in these reports is extremely difficult since most of them are unstructured texts, usually digitized as PDF documents and often including scanned images. To address this issue, a working group formed by text mining experts and toxicology specialists was created with the intention of implementing a text mining system that is able to identify, capture and standardize findings related to drug treatment (i.e., safety findings) by mining legacy preclinical toxicology reports. The ultimate goal of this effort is to develop text mining tools to (semi-)automatically populate the eTRANSAFE Preclinical Database. Importantly, these tools would not replace human experts, but support and facilitate the identification of safety findings that can be later validated by experts, thus expediting the overall process. The knowledge extracted from the textual contents of toxicology reports is designed to provide valuable insights to support the in silico modelling activities that are developed within the project. 

**Figure 5:**
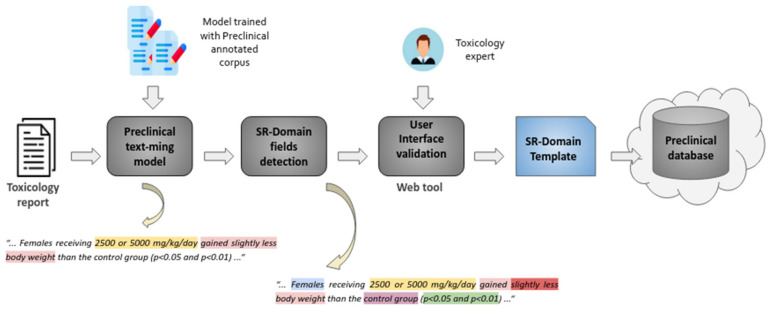
Overview of the preclinical text mining pipeline.

Figure 5 provides an overview of the text mining pipeline devised to extract, characterize and validate safety findings by analysing toxicology reports. Properly trained text analysis models are used to extract the safety information of interest. This is achieved by processing the contents of toxicology reports so as to detect the diverse key features that contribute to characterizing each safety finding, i.e., type, dose, sex, group, etc. Then, by means of a web interface, toxicology specialists can easily validate and refine the information automatically extracted so as to populate a database of safety findings.

A core activity useful to enable the automated extraction of information from toxicology reports has been the creation of a corpus of preclinical toxicology reports where safety findings have been manually annotated. This corpus, curated thanks to the contribution of 13 toxicological experts, includes more than 3000 sentences extracted from a collection of 181 preclinical toxicology reports donated by pharmaceutical companies in eTRANSAFE. Toxicological experts analysed the textual contents of the preclinical reports and annotated safety findings by spotting the text excerpts that characterized a varied set of facets of each finding, including the type of finding, the group of animals and the dose of the drug at which the finding was observed. The creation of such a corpus required the addressing of a variety of aspects, from legal issues related to the access to the legacy reports that would be part of the corpus, to more operational ones such as the establishment of a group of curators, the development of annotation guidelines, the definition of an annotation schema and the adaptation of an annotation tool to support the remote curation process. The close collaboration and communication between text mining and toxicology experts was essential to the success of this endeavour. The annotations included in the corpus are exploited to train and evaluate the text analysis models integrated in the text mining pipeline.

## 6. ToxHub Concept and Architecture

**Figure 6:**
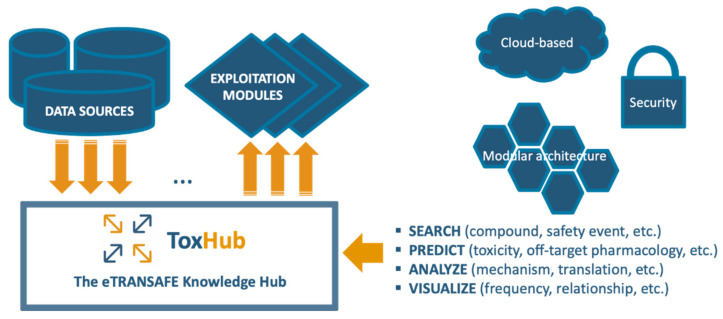
The eTRANSAFE ToxHub concept.

The eTRANSAFE project has developed a software platform based on a knowledge hub, so called ToxHub, which centralizes the access to all data sources and exploitation modules (Figure 6). ToxHub has a modular architecture consisting of a collection of independent components hosted in a secure cloud as a Kubernetes cluster [15]. This solution is extremely flexible and can be deployed in different ways: the whole system can be hosted by a commercial cloud provider or a private instance can be installed locally in the computational facilities of the final user (e.g., a pharmaceutical company). This flexibility would allow a route to overcome the restrictive policies imposed by pharmaceutical companies to guarantee the confidentiality of sensitive information.

**Figure 7:**
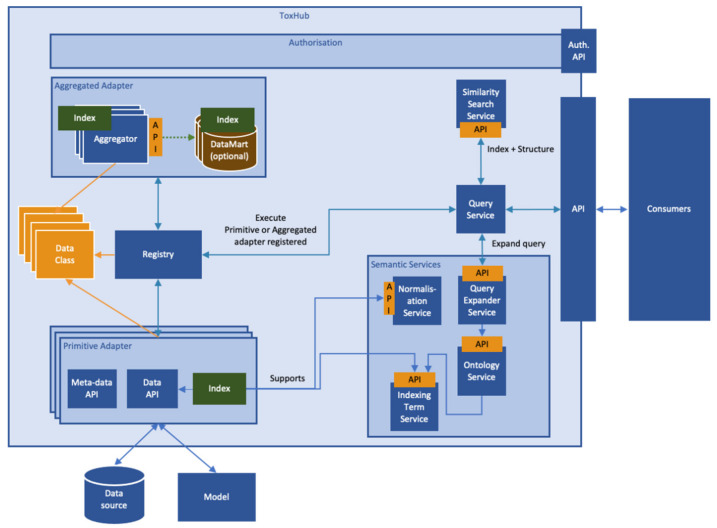
Logical architecture of the eTRANSAFE software platform (ToxHub).

ToxHub components are containerized applications which communicate using appropriate Application Program Interfaces (APIs). A high-level description of the system logical architecture, showing the most important components and their relationships, is depicted in Figure 7.

Some of the components (exploitation modules) provide web-based graphical interfaces which allow, for example, querying the data sources, visualizing the data resulting from the queries or predicting compound properties, directly from an Internet browser.

This modular architecture imposes the use of an abstraction layer wrapping heterogeneous data sources to allow simultaneous querying of some or all of them. This layer is the so-called primitive adapter (Figure 6), which provides a single API for accessing all the data sources, returning data structured in a consistent data-class. This solution, for example, will allow extraction of data from preclinical and clinical data sources in formats suitable for integrated data analysis and visualization.

## 7. Modelling Tools in eTRANSAFE: The Flame Tool

The eTRANSAFE ToxHub was conceived as a one-stop shop with integrated access to predictive models. The component we have developed for supporting the modelling tasks is Flame, an advanced modelling framework for the development and hosting of in silico models, suitable for being used in production environments as a prediction service.

**Figure 8:**
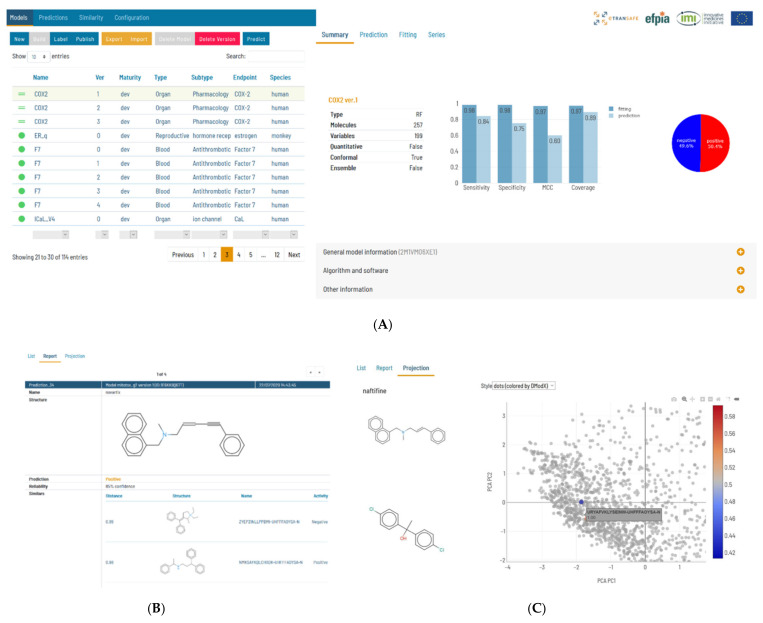
(**A**) Snapshot of the Flame graphical user interface showing on the left-hand side the model list and on the right-hand side information on the selected model, in this case a classifier. The selected tab presents an initial overview of the model quality and the proportion of positive and negative values. (**B**) A view of the prediction result that shows the compound structure, the prediction result and the associated confidence. The structure of the nearest compounds present in the training series is shown as a list. (**C**) In this view of the prediction results, the predicted compounds are represented in a PCA scores plot, together with the training series. The structures of the compounds are shown on mouse hover. The dots of the predicted compounds are coloured according to the distance to the model (DModX).

Flame is a web application with a backend written in Python, implementing a wide panel of predictive modelling functionalities and a modern frontend written in Angular [16], allowing easy access to all the framework functionalities (Figure 8A). This architecture allows the use of Flame remotely as a ToxHub component accessible to all eTRANSAFE partners, but also allows it to be installed locally as a desktop application or in the company intranet to be used as a private prediction server. Models developed using Flame are encapsulated in self-contained predictive engines that can be easily transferred between Flame instances. This facilitates collaboration between academic and industrial partners, since models generated using open data can be easily distributed and used in production in their original form or enriched by retraining them locally with confidential data.

Flame allows the building of models nearly automatically, starting from a training series which can be entered as a single annotated SDFile. It implements natively a wide variety of machine learning methods (Random Forest, XGBOOST, Partial Least Squares, Support Vector Machine and Gaussian Naïve Bayes, all of them as regressors or classifiers) [17] as well as standardization workflows (e.g., EBI workflow [18] or standardizer [19]) and molecular descriptors (RDKit properties and molecular descriptors) [20]. All these methods are based in robust open-source libraries, but proprietary tools can be easily integrated because the source code has been designed to support them as external tools. In Flame, the prediction output can be used as input for other models, allowing the models to be combined in a flexible way (majority voting, consensus, mean, median, etc.). This combination can be easily customized to create rules and combine hazard with exposure.

In our view, the final aim of predictive models is to present prediction results which can help users to make decisions, together with other sources of evidence. This means that presented model results must be accompanied with information about their reliability and practical interpretation. The native implementations of machine learning methods support a conformal framework [21], thus providing rigorous uncertainty estimations expressed as probabilistic confidence intervals. The Flame Graphic User Interface (GUI) was designed to be user-friendly and show relevant information with different levels of detail: from model quality as summaries, to 2D Principal Component Analysis (PCA) projections of the training series showing the compound structures, and the distribution of the values in the training series. Prediction results show the values, units and the interpretation for each individual result, together with individual uncertainty estimations that incorporate the model applicability domain. We also show the structures and properties of the closer compound in the training series (Figure 8B) and 2D projections on the training series 2D spaces, obtained by PCA of the model descriptors, representing as well as the distance to the model (Figure 8C)

Flame is fully open-source and can be used as a standalone component. The source code is accessible at https://github.com/phi-grib/flame, where we provide instructions for using the Python library directly (e.g., using a Jupyter notebook), from a terminal, integrated in scripts, making calls to the REST API or using the GUI described above. Installers for Windows and Linux operative systems are also freely distributed.

## 8. Mechanistic Modelling in eTRANSAFE

The need to move towards a mechanistic-based risk assessment is widely recognized, since it will strengthen the causality connection between compound exposure and the occurrence of adverse outcomes [22]. Systems Toxicology approaches, based on network science and machine learning and embracing different types of data (omics as well as clinical and biomedical data), are especially suited to the study of the perturbations elicited by drugs within the context of cellular networks and in this way provide insight into the molecular mechanisms leading to drug adverse outcomes. 

Network science approaches have been widely used across different domains, from life sciences to medical and clinical research [23,24]. They rely on a simple observation: genes or proteins involved in biological processes underlying the proper functioning of the organism rarely act in isolation but as part of networks of interactions between biomolecules. Therefore, modelling the relationships between biomolecules helps to characterize the impact of perturbations in the human body. In fact, previous studies showed that biomarkers identified using cellular network information are more reproducible than individual marker genes and achieve higher accuracy in the classification of disease states [25]. Furthermore, toxicity pathways tend to be more conserved across species than individual genes, therefore supporting a translational safety assessment [26]. 

**Figure 9:**
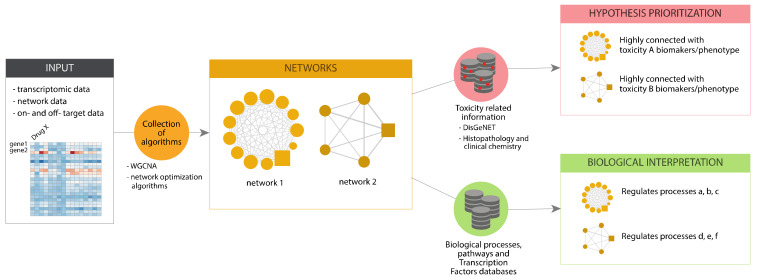
Scheme of the pipeline for toxicogenomics analysis implemented in eTRANSAFE.

In eTRANSAFE, we have implemented different approaches based on Systems Toxicology principles to identify toxicity pathways as key processes between a molecular initiating event and an adverse outcome [27]. These approaches integrate a wide scope of omics data (genomics, transcriptomics, proteomics, interactomics) with information on drug targets, off-targets and pathological outcomes to deliver mechanistic hypotheses of the adverse outcomes elicited by drugs (the toxicity pathways). These approaches, schematized in Figure 9, are based on graph optimization on the human interactome and signalling pathways or correlation patterns in large transcriptomics/toxicogenomics datasets. They use as a starting point different omics and biological data, and by applying network optimization algorithms or gene correlation network methods, identify network modules associated to a particular Molecular Initiating Events (MIE) or the subsequent Key Events (KE). Then, these network modules can be further analysed to learn more about the biological processes that they represent, by means of gene enrichment analyses using different biological annotations followed by interpretation (Biological Interpretation), as well as with information on adverse outcomes and pathology biomarkers to identify those network modules associated with the toxicity endpoint (Hypothesis Prioritization).

**Figure 10:**
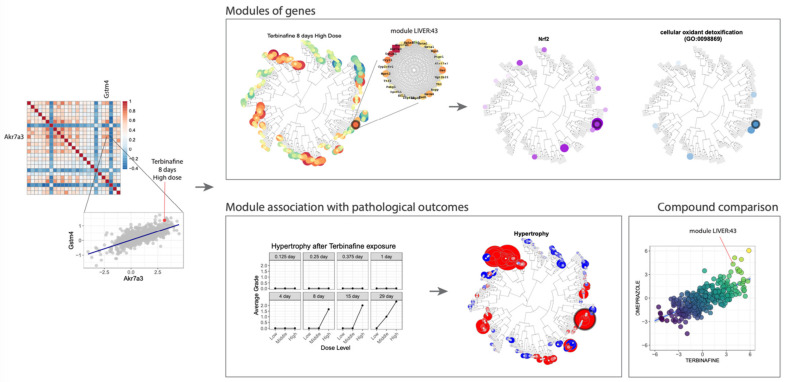
An example of a toxicogenomics analysis carried out in eTRANSAFE using the TXG-MAPr tool.

One example of such an approach is the TXG-MAPr tool that is focused on transcriptomic data, which are quite abundant in the field of toxicogenomics [28]. The TXG-MAPr allows dimensionality reduction and improved interpretation of in vivo transcriptomic data (Figure 10) and is based on a widely applied network analysis algorithm [29]. Leveraging large toxicogenomic databases, such as TG-GATEs [30], groups of co-regulated genes (modules) can be identified, pinpointing known pathways and suggesting new processes involved in drug toxicity. Whenever toxicogenomic data are not available for a compound, alternative methods based on network optimization algorithms on the human interactome are a suitable alternative for the identification of toxicity pathways. All in all, these approaches offer complementary perspectives, one based on transcriptomic data and the other on proteomic data, that can even be combined to infer toxicity pathways.

These data-driven, mechanistic approaches have the advantage of first being able to capture new biomolecular interactions that participate in response to toxicants, which are not currently collated in resources that provide information about canonical pathways. Second, the pathway space has higher predictive robustness than models in the gene space [31] (Segura-Lepe 2019). Finally, the third advantage is that these data-driven approaches would be capable of providing a complete map of the full spectrum of toxicity pathways that underlie adverse events [32,33,34].

## 9. Opportunities Opened by eTRANSAFE: The eTRANSAFE Virtual Control Group 

The pharmaceutical companies participating in eTRANSAFE have developed individual decision trees to identify which data can be shared (i.e., data which are “non-confidential”) and data which have to remain confidential. Though the decisions may vary from company to company, it became evident that the most sensitive information in datasets or studies is the chemical structure, disclosure of the target or indication and, in some cases, specific toxicological findings, particularly when these are rare or can be related to the pharmacological mode of action. On the other hand, there was a common understanding that some data can be shared without any restriction because they cannot be linked back to an individual test item or mechanisms. This particularly applies to data from control animals. Sharing data from control animals opens the door for completely new analyses and approaches to spare animals in studies. The shared control animal data can be used to construct so-called “Virtual Control Groups” (VCG) or replace real control animals by virtual ones [35].

The prerequisite for such an approach is a well curated repository and a thorough understanding of which factors in the dataset influence variability. In the field of carcinogenicity studies, such data collections and analyses are common to assess the background incidence of tumours [36]. For systemic toxicity studies, such an approach has not yet been applied. The eTRANSAFE project has started to collect control data from oral 4-week repeated dose toxicity studies in rats from five companies. Essentially, all routine endpoints covered in an oral repeated dose toxicity study are included, namely study definition, animal data, clinical chemistry, haematology, urinalysis, organ weights, gross pathology and histopathology findings (see Table 2).

Many of the parameters collected are continuous data, but others, e.g., those from histopathology, are scientific opinion-based discrete gradings derived from a visual examination and obtained after expert evaluation. The continuous data will be analysed by descriptive statistics for their mean, distribution and variability. The discrete gradings will be evaluated using nonparametric methods. Here, the incidence of findings and changes in incidences will provide insights for deciding if an observation is a treatment-related effect or still in the range of control group animals.

The eTRANSAFE VCG database supports a quantitative assessment that may provide a complete picture of the variability of given historical control data not only for single laboratories but on inter-laboratory comparisons. These analyses will be performed first on an intra-company basis and then on an inter-company basis, with the objective of gaining a better understanding of which thresholds need to be set in the variability for using the data in the virtual control approach, but also to detect factors which generate variability (e.g., can rats of the same strain and from the same breeder be used even though they have been used at different sites?). 

Particularly, the analysis and use of historical negative control data is essential for judging both quality and proficiency control in toxicological assays. It will provide important instruments for data evaluation and interpretation of current experiments. The negative control data are being collected and continuously used to evaluate the validity of experiments by comparing the historical control data with the current negative controls. Further analysis of treatment-related findings requires robust historical data reflecting the experimental conditions used for the evaluation. For this purpose, the quality of the control data and the adequacy in the statistical evaluation are of the highest relevance. 

The ultimate goals of the use of the eTRANSAFE VCG database are the reduction of the number of control group animals or a complete replacement of the control group by virtual control animals based on data drawn from the database. The prerequisite to achieve these goals is a thorough understanding of the data variability for the laboratory performing the studies and clear rules and definitions for thresholds of variability in order to adequately safeguard the sensitivity and specificity of the in vivo studies with regard to the question of whether an observation is treatment-related, i.e., compound-related, or not.

The VCG activity probably will not be fully accomplished during the course of the eTRANSAFE project. It is expected that internal data collection in qualification within individual companies will take at least one year. Thereafter, a cross-company validation exercise may be started, and the results presented to regulatory authorities. However, the pharmaceutical companies can already use the collected, curated and analysed data for the non-GLP studies (e.g., virtual control groups could be used for dose-range-finding studies without any formal approval by the authorities). It is indeed expected that the VCG activity will be part of the sustainability of the eTRANSAFE project and that the VCG database and adjacent statistical tools will be continuously updated by at least a group of partners constituting a task group after the end of the project. Overall, it is expected that the full use of VCGs will take a number of years of parallel work with real control groups, before health authorities could validate this approach and accept its incorporation into regulatory guidelines.

## 10. Conclusions and Prospects

The IMI eTRANSAFE project, building on the achievements of its predecessor, eTOX, is addressing key needs and gaps in pursuit of a more holistic assessment of drug toxicity. The translational perspective is increasingly seen as essential to both be able to improve the predictive potential of animal studies and to better understand adverse events observed in humans. Integrative exploitation and expanded availability of preclinical and clinical data is possible; however, a multitude of technical, legal, ethical, financial, cultural and psychological obstacles require a concerted approach to be overcome, and eTRANSAFE represents the type of collegiate strategy that can pave the way for future progress. Exploration of data and read-across strategies can be complemented with predictive modelling and biological substantiation approaches that offer a deeper view on potential toxicity and plausibility of observed effects; in other words, holistic views need not only to refer to data types and coverage, but also to types of perspectives that can be harmonically integrated into the daily workflows of the drug discovery and development life cycle.

Two thirds through its planned duration, eTRANSAFE has already demonstrated the feasibility of a holistic approach to toxicity assessment, collecting otherwise inaccessible data from private companies, facilitating a translational linkage between the preclinical and clinical worlds, and complementing the exploitation of such data with predictive modelling and biological plausibility strategies and tools, as well as promoting innovative approaches such as the development of virtual control groups or advanced text mining approaches. To our knowledge, it also represents the first attempt to systematically map semantic concepts between preclinical and clinical data related to toxicity. The open architecture implemented in the eTRANSAFE system is expected to facilitate its maintenance and development, and to flexibly allow the addition of supplementary data sources and tools to expand its scope and features. The eTRANSAFE system is expected to change the existing workflows in toxicology to help to provide better drugs faster, including the drug repurposing operations. Such a system is foreseen as the core element of a future ecosystem that unites end users and developers for an ever-expanding in silico framework for translational safety assessment. This will depend on successfully creating value propositions and incentives that attract a critical mass of tool and model developers both from academia and industry, which will amplify the potential impact of eTRANSAFE to enable a true quantum leap in the field.

## Figures and Tables

**Table 1:** Objectives of the eTRANSAFE project.

Item	What	Why
**Preclinical** **database**	Collect and build the largest existing preclinical database/repository (including external data sources).	Necessary for data mining, modelling and translational assessment.
**Clinical** **database(s)**	Collect as much human study data as possible (clinical trials, PSURs, etc.).	Necessary for data mining, modelling and translational assessment.
**Translation**	Build a computational system to allow the systematic assessment of animal data for their validity and value in human safety.	Looking into details, species by species, organ by organ, toxicity by toxicity, target by target, where animals are relevant and where not, to anticipate potential human safety outcome. Potential to modify the future way of running preclinical safety assessment.
**Data mining** **and analysis**	Build query tools for joint data retrieval and mining in several databases/repositories.	Read-across analysis, finding precedents to user cases.
**Graphic**	Establish efficient data visualization, zooming in on essential.	Speed and ease of analysis. Efficient communication. Toxicology report input.
**Toxicological** **models**	Explanatory and predictive in silico models build on high quality data.	Improve relevance of models to match the druggable chemical space used in pharma. Emergence of Predictive Safety in pharma, which needs reliable models built using collective pharma history.
**Policies**	Formulate principles and rules for data sharing and model validation.	Facilitate current and future initiatives of data transparency and precompetitive data sharing.
**Sustainability**	To assure continuity and potentially commercial viability after end of project.	Pharmaceutical companies (and other parties) will want to continue using the system after end of the project.

**Table 2:** Measurements and observations performed in an oral 4-week repeated dose toxicity study in rats, which were collected for the eTRANSAFE Virtual Control Group (VCG) database.

Category	Measured or Observed Parameters
Study definition	Route or administration, duration, test facility
Animal data	Species, strain, source, age, sex, initial body weight, body weight gain
Clinical chemistry	Sodium, potassium, calcium, chloride, inorganic phosphate, glucose, urea, creatinine, total bilirubin, cholesterol, triglycerides, bile acids, total protein, albumin, albumin/globulin ratio, alanine aminotransferase, aspartate aminotransferase, alkaline phosphatase, glutamate dehydrogenase
Haematology	Erythrocytes, haemoglobin, haematocrit, mean cell volume, mean haemoglobin content, mean haemoglobin concentration, platelets, reticulocytes, leukocytes, neutrophilic granulocytes / (%) and (10^3^/µL), lymphocytes/(%) and (10^3^/µL), eosinophilic granulocytes/(%) and (10^3^/µL), basophilic granulocytes/(%) and (10^3^/µL), monocytes/(%) and (10^3^/µL), large unstained cells/(%) and (10^3^/µL), prothrombin time/(s), activated partial thromboplastin time/(s)
Urinalysis	pH value, protein (grading), glucose (grading), bilirubin (grading), blood (grading), ketone (grading), sediment, specific gravity, urine weight (g)
Organ weights	Liver, heart, kidneys, spleen, thymus, ovaries, uterus, brain, adrenal glands (both), thyroid/parathyroid, testes, prostate, seminal vesicles, epididymites
Histopathology	Adrenal gland, aorta, bone, bone marrow, brain, oesophagus, eye, heart, intestine large (cecum), intestine large (colon), intestine large (rectum), intestine small (duodenum), intestine small (ileum), intestine small (jejunum), kidney, knee joint, liver, lung, lymph node (mandibular), lymph node (mesenteric), mammary gland, muscle (skeletal), nerve (optic), nerve (sciatic), pancreas, parathyroid gland, Peyer’s patches, pituitary gland, reproductive organs (male, epididymis), reproductive organs (male, prostate), reproductive organs (male, seminal vesicle), reproductive organs (male, testis), salivary gland, skin, spinal cord, spleen, stomach, thymus, thyroid gland, trachea, ureter, urinary bladder, reproductive organs (female, ovary), reproductive organs (female, oviduct), reproductive organs (female, uterus), reproductive organs (female, vagina), gallbladder (mouse specific), larynx (fixation only)
